# Validation of ethnopharmacological uses of *Heliotropium strigosum* Willd. as spasmolytic, bronchodilator and vasorelaxant remedy

**DOI:** 10.1186/s12906-015-0697-1

**Published:** 2015-06-06

**Authors:** Khalid H. Janbaz, Sana Javed, Fatima Saqib, Imran Imran, Muhammad Zia-Ul-Haq, Vincenzo De Feo

**Affiliations:** Faculty of Pharmacy, Bahauddin Zakariya University, Multan, Pakistan; Department of Pharmacognosy, University of Karachi, Karachi, Pakistan; Department of Pharmacy, University of Salerno, Fisciano, Salerno Italy

**Keywords:** *Heliotropium strigosum*, Spasmolytic activity, Bronchodilator activity, Vasorelaxant activity

## Abstract

**Background:**

*Heliotropium strigosum* is used in traditional medicine to manage gastrointestinal pain, respiratory distress and vascular disorders. The present study was undertaken to provide scientific evidences for these folkloric uses by *in vitro* experimental settings.

**Methods:**

A crude methanol extract of the *Heliotropium strigosum* (Hs.Cr) was tested *in vitro* on isolated rabbit jejunum preparations to detect the possible presence of spasmolytic activity. Moreover, isolated rabbit tracheal and aorta preparations were used to ascertain the relaxant effects of the extract.

**Results:**

The Hs.Cr exhibited relaxant effects in rabbit jejunum in a concentration dependent manner (0.01-3.0 mg/ml). The Hs.Cr also relaxed K^+^ (80 mM)-induced spastic contractions in rabbit jejunum and shifted the Ca^2+^ concentration response curves towards right. The extract relaxed carbachol (1 μM)- as well as K^+^ (80 mM)-induced contractions in rabbit trachea at concentrations ranging from 0.01 to 10 mg/ml. Moreover, Hs.Cr. also relaxed (0.01-3.0 mg/ml) the phenylephrine (1 μM)- and K^+^ (80 mM)-induced contractions in isolated rabbit aorta.

**Conclusions:**

The Hs.Cr was found to exhibit spasmolytic, bronchodilator and vasorelaxant activities on isolated rabbit jejunum, trachea and aorta preparations, likely mediated through Ca^2+^ channel blockade. This finding may provide a scientific basis for the folkloric uses of the plant.

## Background

*Heliotropium strigosum* Willd. (Boraginaceae), locally known as *Kharsan, Gorakh pam* and *Bhangra*, is a herb widely distributed throughout Pakistan [1]. It grows in arid places, land rocks, hypersaline soils and regions where ecological environment is markedly unkind, due to the high temperature and reduced carbon availability. The herb is perennial, prostrate to procumbent, branched and woody at the base. The leaves are 1.3-2.5 cm long, linear-lanceolate, acute, strigosely hairy on both surfaces and becoming smaller upwards. The flowers are white in color. The inflorescence is bracteate and simple or paired. The calyx-lobes are ovate, acute, and enlarged into fruit. The corolla is slaver shaped and stigma is narrowly conical. The fruit is depressed at the apex up to 4 mm, more or less united, hairless. The plant flowers from March to October [2, 3]. The plant is traditionally used by Thari people of Nara desert in Pakistan in multiple gastrointestinal disorders, as a diuretic and a laxative. The juice of the plant is applied to treat sore eyes, gum boils and sores [4]. The plant is used to treat abscess of breast by applying a blend of the whole herb with butter. The plant is also claimed to be effective in treating scorpion stings, snake bite, wounds and ulcers. The infusion of the herb is claimed to possess “cooling effect” [5]. Furthermore, the plant is also claimed to be effective in renal, muscular and gynecological disorders and is also used to relieve body discomfort [6].

Strigosine, a pyrrolizidine alkaloid, has been isolated from *H. strigosum* [7]. Methanolic extracts of the plant showed cytotoxic and phytotoxic activities [8]. Recently, antioxidant and antimicrobial activities have also been reported [9]. The ethyl acetate fraction of a crude extract of *H. strigosum* exhibited antipyretic activity [10]. Although used as a remedy for multiple ailments like airway, gastric and cardiovascular ailment, *H. strigosum* is a very less-researched plant, as no detailed pharmacological studies exist.

Smooth muscle play a critical role in regulating functions of the gastrointestinal (GIT), respiratory and cardiovascular (CV) systems. Any irregularity in smooth muscle contractility ultimately results in many complication for GIT, including spasmogenicity of the small intestine, abdominal cramps and diarrhea. In CV, the irregular muscle contractility can determine hyper- and hypotension, and in respiratory system airway disorders like asthma. In all above mentioned disorders, cytosolic Ca^2+^ is a key regulator in the modulation of smooth muscle activity and the sensitivity to Ca^2+^ of the contractile elements changes depending on the environment surrounding the cell [11].

To study the possible spasmolytic, vaso- and tracheal relaxant/constrictive effects of a plant extract, a tissue system comparable to human body is needed. Usually, isolated tissues from guinea pig, rabbit or rat are used to evaluate the effects of a drug on the GIT, the CV and the respiratory systems, as these animals shares many anatomical and physiological similarities of human organs. Isolated tissue are preferred over intact animals, as many experiments on isolated tissues permit easy of handling, broad visibility, requirement of small amounts of drug and low possibility of interferences in absorption and metabolism. In fact, rabbit jejunum, duodenum, aorta and trachea are preferred tissue strips for their availability, sensitivity, reproducibility of results and cost-effectiveness. Many studies are available in the scientific literature on isolated smooth tissue muscles of different animal models [12–14].

As a part of studies aimed to validate traditional uses of medicinal plants [15, 16], the current study was designed to investigate the therapeutic potential of *H. strigosum* in cardiovascular, respiratory and gastrointestinal ailments.

## Methods

### Plant material

*Heliotropium strigosum* was collected from Khanewal Punjab, Pakistan in September, 2011. The plant was identified by the taxonomist, Dr. Saima Shehzadi, at the Institute of Pure and Applied Biology, Bahauddin Zakariya University, Multan where a voucher specimen (P.Fl. 591-2) was deposited.

### Extraction

The whole plant was shade dried for 10 days. The dried material (1.200 kg) was coarsely ground and soaked in 80 % aqueous-methanol in an amber colored container. This material was retained at room temperature for 7 days with occasional shaking. The material was passed through a double layered muslin cloth and the fluid was subsequently filtered through a Watman-1 filter paper. The extraction procedure was repeated twice and the extracts were combined and evaporated under reduced pressure, to give a dark brown residue with a yield of 9.8 %. The extract was stored at -20 °C and fresh dilutions were prepared on the day of tests.

### Chemicals and drugs

All the chemicals, solvents, and drugs used were of reagent analytical grade. Acetylcholine, carbachol, verapamil chloride and potassium chloride were obtained from Sigma Chemicals Co., St Louis, MO, USA. Methanol, EDTA, glucose, magnesium chloride, magnesium sulfate, phenylephrine, potassium dihydrogen phosphate, sodium bicarbonate, sodium dihydrogen phosphate and calcium chloride were purchased from Merck, Darmstadt, Germany. Sodium chloride, ammonium hydroxide and sodium hydroxide were obtained from BDH laboratory supplies, Poole, England.

### Animals

Adult rabbits (1.0–1.5 kg) of either sex used in experiments were purchased from the local market and housed in animal house of the Faculty of Pharmacy, Bahauddin Zakariya University, Multan. The animals were provided with standard feed and tap water *ad libitum* and were maintained in controlled conditions and 25 °C. The animals were subjected to overnight fasting prior the day of experiments, but had free access to water. All experiments were performed according to the rules of the Institute of Laboratory Animals Resources, Commission on Life Sciences, National Research Council [17] and authorized by the Ethical Committee of the Bahauddin Zakariya University, Multan (EC/01/2011 dated 16.02.2011).

### *In-vitro* experiment on isolated tissues

#### Isolated rabbit jejunum preparation

The methanolic extract of *Heliotropium strigosum* (Hs.Cr) was tested for the possible presence of either spasmolytic or spasmogenic activity by using isolated rabbit jejunum preparations. Isolated rabbit jejunum segments of approximately 2 cm in length were suspended in isolated tissue baths containing Tyrode’s solution, at 37 °C, aerated with carbogen (95 % O_2_ and 5 % CO_2_). The composition of the Tyrode’s solution (mM) was: KCl (2.68), NaCl (136.9), MgCl_2_ (1.05), NaHCO_3_ (11.90), NaH_2_PO_4_ (0.42), CaCl_2_ (1.8) and glucose (5.55). A preload tension of 1 g was applied and intestinal responses were recorded through an isotonic transducer by a Power Lab Data Acquisition System (AD Instruments, Sydney, Australia) attached to a computer installed with a Lab Chart Software (Version 7).

The tissues were allowed to equilibrate for at least 30 min prior to the addition of any drug. Isolated rabbit jejunum preparations exhibit spontaneous rhythmic contractions and allow testing of the antispasmodic (relaxant) effect without application of an agonist [14, 15]. The observed response of the test material was quantified by the application of doses in a cumulative fashion. The relaxant effects of the tested substances were taken as the percent change in spontaneous contractions of the preparation recorded immediately before the addition of the substances.

The possible mechanisms of the relaxant activity of the extract were investigated through the relaxation of the observed sustained spasmodic contractions following the exposure to high concentration of K^+^ (80 mM) [18]. The extract was applied in a cumulative manner to the sustained contractions to achieve concentration-dependent inhibitory responses [19]. The observed relaxant effects on K^+^ (80 mM)-induced contraction was expressed as the percent of the control contractile response.

The calcium channel blocking effect of the test substances were confirmed by previously reported methods [14, 15, 19]. The isolated rabbit jejunum preparations were allowed to stabilize in normal Tyrode’s solution, which was subsequently replaced for 30 min with Ca^2+^-free Tyrode’s solution to which EDTA (0.1 mM) was added in order to remove calcium from the tissues. This bath solution was further replaced with K^+^-rich and Ca^2+^-free Tyrode’s solution, having the following composition (mM): KCl (50), NaCl (91.04), MgCl_2_ (1.05), NaHCO_3_ (11.90), NaH_2_PO_4_ (0.42), glucose (5.55) and EDTA (0.1). Subsequent to an incubation period of 30 min, cumulative Ca^2+^ concentrations were applied to the tissue bath to obtain control calcium dose-response curves (DRCs). On achievement of the super-imposable control calcium dose-response curves (usually after two cycles), the tissues were then washed and allowed to equilibrate with the plant extract for 1 h and then the concentration response curves of Ca^2+^ were recorded and compared to the control curves. The DRCs of Ca^2+^ were recorded in the presence of different concentrations of the plant extracts in tissue bath.

#### Isolated rabbit tracheal preparations

The rabbit tracheas were dissected out and kept in a Krebs solution of the following composition (mM): NaCl (118.2), NaHCO_3_ (25.0), CaCl_2_ (2.5), KCl (4.7), KH_2_PO_4_ (1.3), MgSO_4_ (1.2) and glucose (11.7). The trachea was cleaned free from the surrounding fatty tissues and rings of 2-3 mm width containing 2-3 cartilages were prepared. Each ring was opened by a longitudinal incision on the ventral side opposite to the smooth muscles layer to form a strip with smooth muscles layer in middle and cartilages on both sides. These tracheal preparations were mounted in 20 ml organ bath containing Krebs solution being maintained at 37 °C and aerated with carbogen. A preload tension of 1 g was applied and tissue preparations were allowed to be equilibrated for 1 h prior to any challenge by the extract. Tissue preparations were stabilized by repeated applications of carbachol (1 μM) until constant responses were recorded. The carbachol (1 μM) and high K^+^(80 mM)-induced sustained contractions were subsequently used for testing of different doses of the test material in a cumulative fashions. The isometric responses were recorded through a Power Lab Data Acquisition System (AD Instruments, Sydney, Australia) attached to a computer with a Lab Chart Software (Version 7). The standard drug with Ca^2+^ channel blocking effect, verapamil, was tested on high K^+^(80 mM)- and carbachol- induced spastic contractions in order to confirm the possible mechanism of action.

#### Isolated rabbit aorta preparation

The effect of Hs.Cr on systemic vascular resistance was assessed on isolated rabbit aorta preparations. Rabbits of either sex were sacrificed by a blow on the back of head and descending thoracic aorta was dissected out and kept in the normal Krebs solution. It was then cut vertically in 2-3 mm width segments. Each isolated tissue segment was then hung in a tissue organ bath (Radnoti Organ Bath System, AD Instruments, Sidney, Australia) containing Kreb’s solution aerated with carbogen at 37 °C. A preload tension of 2 g was applied to each preparation and allowed to equilibrate for 1 h. After equilibration, tissue was stabilized by repeated exposure to K^+^ (80 mM) or phenylephrine (1 μM) depending upon the protocol of the experiment. The vasorelaxant/vasoconstrictive effects of the extract were studied in a cumulative manner. Changes in isometric tension of aortic rings were obtained via a force-displacement transducer (Model FORT100, WPI, USA) coupled to a Power Lab data acquisition system (AD Instruments, Sydney, Australia) and a computer running Lab Chart software (version 7).

### Statistical analysis

In isolated tissue experiments, data were expressed as the mean ± SEM and the EC_50_ values with 95 % CI were calculated by using the computer software “Graphpad Prism Program” (version 5.0), San Diego CA, USA. Concentration response curves were analyzed by the non-linear regression of sigmoid response curve (variable slope). The statistic applied was the student’s t- test and p <0.05 was considered as significant.

## Results

### Effect on isolated rabbit jejunum preparations

**Fig. 1:**
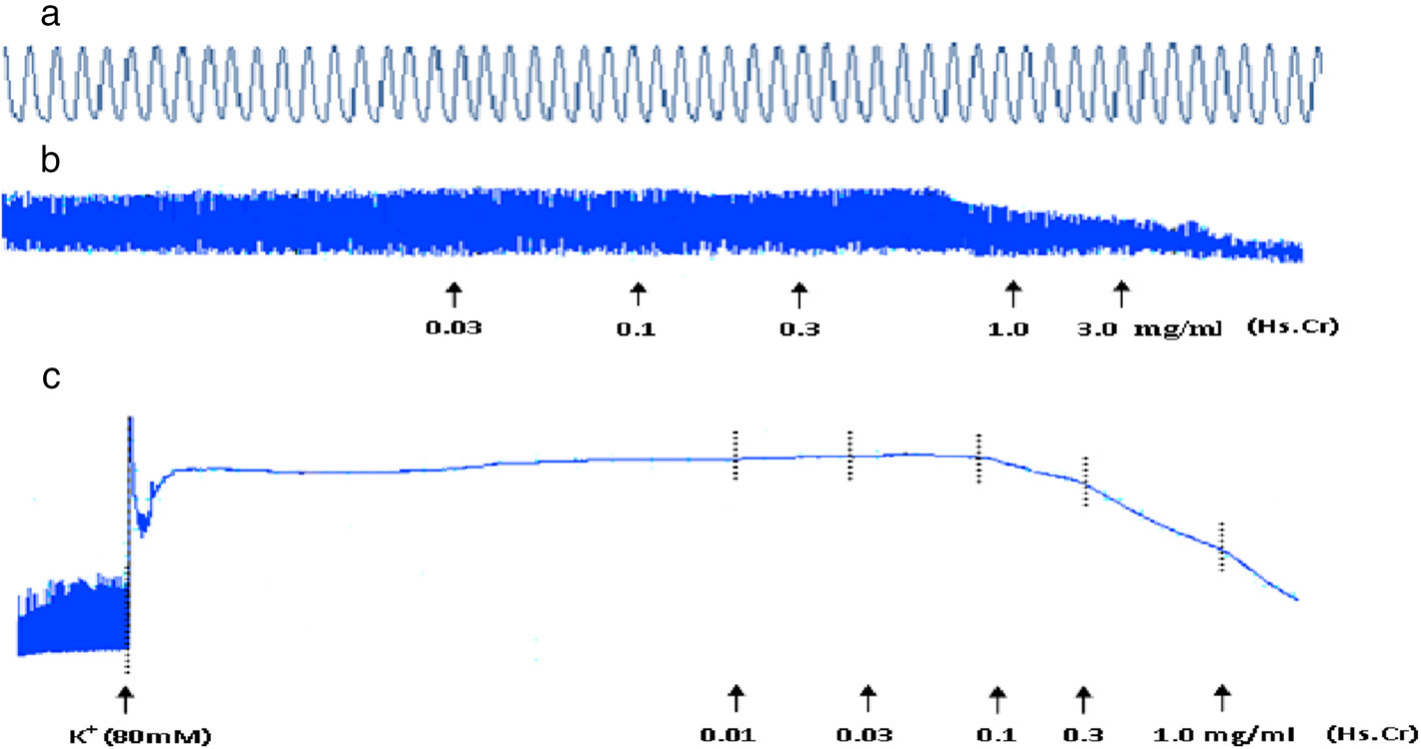
Tracings showing (**a**) the spontaneous contraction of isolated rabbit jejunum and (**b**) the relaxant effect of a crude methanolic extract of *Heliotropium strigosum* (Hs.Cr) on spontaneous- and (**c**) high K^+^ (80 mM)-induced tissue contraction. Hs.Cr was added in increasing concentrations and values listed were the final tissue bath concentrations (n = 5)

**Fig. 2:**
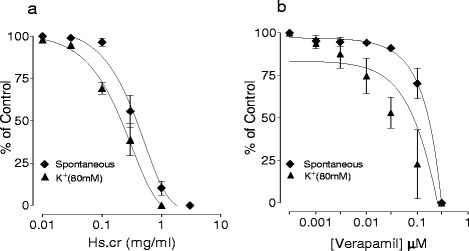
Spasmolytic effects induced in concentration-dependent manner by (**a**) a methanolic extract of *Heliotropium strigosum* (Hs.Cr) and (**b**) verapamil on spontaneous- and high K+ (80 mM)-induced contractions in isolated rabbit jejunum preparations. Values are the mean ± SEM, n = 5

The application of Hs.Cr to spontaneous contractions of isolated rabbit jejunum preparations (Fig. 1b) provoked a concentration-dependent (0.01-3.0 mg/ml) relaxant effect after addition in a cumulative manner; the EC_50_ value was 0.381 mg/ml (95 % CI: 0.043-1.478 mg/ml; n = 5) (Fig. 2a). Similarly, the standard Ca^2+^ channel blocker, verapamil, relaxed the spontaneous contractions in a concentration range of 0.003-1.0 μM, with an EC_50_ value of 0.151 μM (95 % CI: 0.111-0.206; n = 5) (Fig. 2b).

**Fig. 3:**
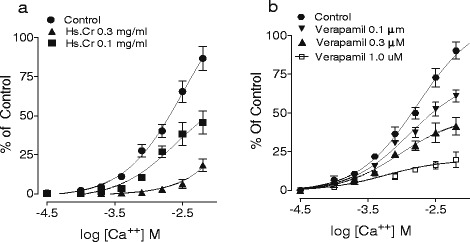
Calcium antagonizing effect of (**a**) a methanolic extract of *Heliotropium strigosum* (Hs.Cr) and (**b**) verapamil on concentration response curves of Ca^2+.^ in isolated rabbit jejunum preparations. Values are the mean ± SEM, n = 5. Control Ca^2+^

Moreover, Hs.Cr caused a relaxation of K^+^ (80 mM)-induced contractions in isolated rabbit jejunum preparations with an EC_50_ value of 0.243 mg/ml (95 % CI: 0.012-0.832; n = 5) (Fig. 1c and Fig. 2a). Likewise, verapamil also relaxed K^+^ (80 mM)-induced contractions with an EC_50_ value of 0.041 μM (95 % CI: 0.025-0.068; n = 5) (Fig. 2b). Furthermore, Hs.Cr caused rightward shifting of Ca^2+^ concentration response curves (CRCs) at a concentration range of 0.1-0.3 mg/ml in a manner similar to verapamil (Fig. 3).

### Effect on isolated rabbit tracheal preparations

**Fig. 4:**
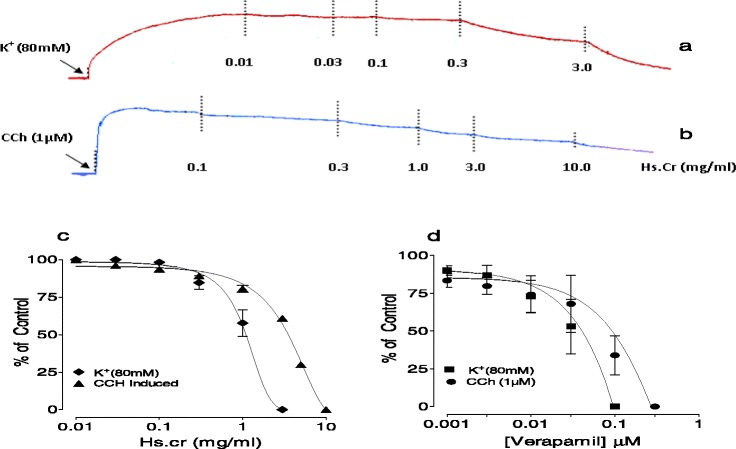
Concentration dependent bronchodilator effect of a methanolic extract of *Heliotropium strigosum* (Hs.Cr) (**a**, **b** and **c**) and (**d**) verapamil on carbachol (CCh: 1 μM)- and high K^+^ (80 mM)- induced contractions in isolated rabbit tracheal preparations. Values are the mean ± SEM, n = 5

The Hs.Cr caused a relaxation of both carbachol (CCh)- and K^+^ (80 mM)-induced contractions in isolated rabbit tracheal preparation with respective EC_50_ values of 3.26 mg/ml (95 % CI: 0.023- 9.783 mg/ml; n = 5) and 1.17 mg/ml (95 % CI: 0.014-2.591 mg/ml; n = 5) (Fig. 4a, b, c). Similarly, verapamil also produced relaxation of carbachol (1 μM)- and K^+^(80 mM)-induced contractions in isolated rabbit tracheal preparations with respective EC_50_ valuess of 0.114 (95 % CI: 0.057-0.227 mg/ml; n = 5) and 0.048 μM (95 % CI: 0.028-0.083 mg/ml; n = 5) (Fig. 4d).

### Effect on isolated rabbit aorta preparations

**Fig. 5:**
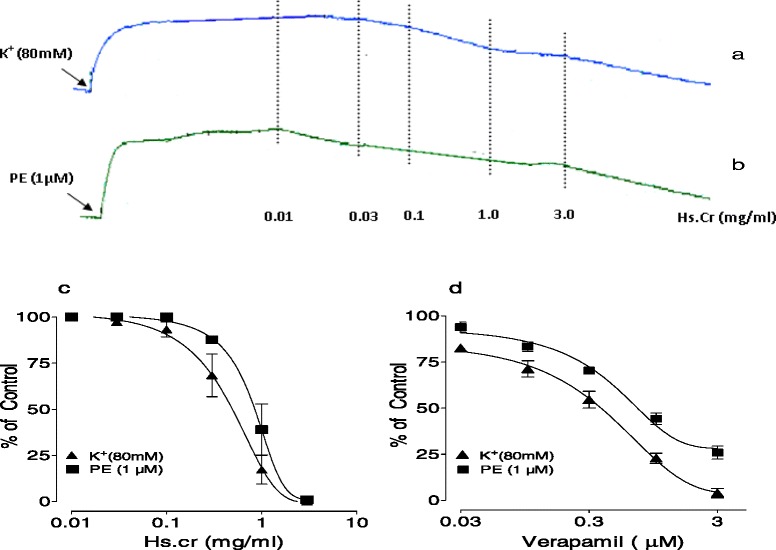
Concentration dependent vasodilator effect of a methanolic extract of *Heliotropium strigosum* (Hs.Cr) (**a**, **b** and **c**) and (**d**) verapamil on phenylephrine (PE: 1 μM)- and high K+ (80 mM) induced contractions in isolated rabbit aorta preparations. Values are the mean ± SEM, n = 5

The Hs.Cr exerted a relaxant effect on phenylephrine (1 μM)- and K^+^(80 mM)-induced contractions in isolated rabbit aorta preparations with respective EC_50_ values of 0.831 mg/ml (95 % CI: 0.102-2.398 mg/ml; n = 5) and 0.493 mg/ml (95 % CI: 0.113-1.782 mg/ml; n = 5) (Fig. 5a, b, c). Similarly, verapamil also produced relaxation of phenylepherine (1 μ M)- and K^+^(80 mM)-induced contractions, with respective EC_50_ values of 0.90 μM (95 % CI: 0.03-5.02 μM; n = 5) and 0.43 μM (95 % CI: 0.03-1.98 μM; n = 5) (Fig. 5d).

## Discussion

In this study we observed the effects of a methanolic extract of *H. strigosum* in smooth isolated muscle by constructing dose-response curves. The application of the extract to the spontaneous contractions of isolated rabbit jejunum preparations resulted in a relaxation of the contractile effect, evident by the decrease in the amplitude as well as the frequency of the contractions. The spontaneous contractions and relaxation of intestinal tissues is based on the phenomenon of alternative depolarization and repolarization. The maximal depolarization results from an action potential generation by the rapid inward Ca^2+^ movement through voltage dependant L-type Ca^2+^ channels (VDLCs) [20]. Several neuro-endocrinal agonists, like acetylcholine, histamine and serotonin, increase the contractile activity of smooth muscle through a rapid increase of the cytosolic Ca^2+^ concentration [11].

The Hs.Cr was applied to K^+^(80 mM)-induced contractions in isolated rabbit jejunum to account for the observed relaxant effect on spontaneous contractions in the same system. The preparations, after exposure to K^+^(>30 mM), are liable to be contracted due to the opening of VDLCs, the rapid influx of extracellular Ca^2+^ and an increase in cytosolic Ca^2+^ [21]. The extract was found to relax K^+^(80 mM)-induced contractions; hence it is possible to hypothesize that it cause the blockade of Ca^2+^ channels [22]. The proposed Ca^2+^ channel blocking activity was confirmed further by the rightward shift of calcium CRCs in isolated rabbit jejunum preparations in a manner similar to verapamil. This drug is a well known member of Ca^2+^ channel blockers, exerting an inhibitory effect on Ca^2+^ influx [23, 24].

To assess the effectiveness of Hs.Cr in respiratory disorders, it was applied on CCh (1 μM)- and K^+^(80 mM)-induced contractions in isolated rabbit trachea. It relaxed both carbachol (1 μM)- and K^+^(80 mM)-induced contractions and this non-specific relaxant effect is presumed to be mediated through Ca^2+^ channel blocking activity [25].

The Hs.Cr was also tested on isolated rabbit aorta pre-treated with phenylephrine (1 μM)- and K^+^(80 mM), as these preparations have been used to investigate the nature of Ca^2+^ channel blocking activity. The K^+^(80 mM)-induced contractions are outcome of Ca^2+^ channels opening, influx of extra-cellular Ca^2+^ and Ca^2+^ release from endoplasmic reticulum. Phenylephrine- induced contractions are mediated through the activation of α-adrenergic receptors and the subsequent Ca^2+^ influx through the receptor operated Ca^2+^ channels (ROCs). Hs.Cr relaxed both phenylephrine and K^+^(80 mM)-induced contractions in a manner comparable to verapamil. The observed nonspecific relaxant effect is likely to be mediated through a Ca^2+^ channel blocking mechanism. The Ca^2+^ channel blockers are reported to provide relief in respiratory congestions and such activity of the Hs.Cr may provide a scientific basis for the traditional use of the plant in respiratory stress.

## Conclusions

This study provides a rationale for the folkloric use of *H. strigosum* in gastrointestinal spasms, respiratory distress and vasospastic disorders, mediated possibly through the blockade of voltage-dependent calcium channels or the release of intracellular Ca^2+^. However, more detailed studies are required to establish the safety, efficacy and toxicity of this plant and to isolate the bioactive constituents.
